# Establishment of Genome Based Criteria for Classification of the Family Desulfovibrionaceae and Proposal of Two Novel Genera, *Alkalidesulfovibrio* gen. nov. and *Salidesulfovibrio* gen. nov.

**DOI:** 10.3389/fmicb.2022.738205

**Published:** 2022-05-25

**Authors:** Mi-Jeong Park, Yun Jae Kim, Myeongkyu Park, Jihyun Yu, Teddy Namirimu, Yoo-Rim Roh, Kae Kyoung Kwon

**Affiliations:** ^1^Marine Biotechnology Research Center, Korea Institute of Ocean Science & Technology, Busan, South Korea; ^2^Department of Applied Ocean Science, University of Science and Technology, Daejeon, South Korea; ^3^Interdisciplinary Program in Bioinformatics, Seoul National University, Seoul, South Korea

**Keywords:** Desulfovbrionaceae, AAI, RSCU, genome, *Desulfovibrio*, taxogenomics, classification criteria

## Abstract

Bacteria in the Desulfovibrionaceae family, which contribute to S element turnover as sulfate-reducing bacteria (SRB) and disproportionation of partially oxidized sulfoxy anions, have been extensively investigated since the importance of the sulfur cycle emerged. Novel species belonging to this taxon are frequently reported, because they exist in various environments and are easy to culture using established methods. Due to the rapid expansion of the taxon, correction and reclassification have been conducted. The development of high-throughput sequencing facilitated rapid expansion of genome sequence database. Genome-based criteria, based on these databases, proved to be potential classification standard by overcoming the limitations of 16S rRNA-based phylogeny. Although standards methods for taxogenomics are being established, the addition of a novel genus requires extensive calculations with taxa, including many species, such as Desulfovibrionaceae. Thus, the genome-based criteria for classification of Desulfovibrionaceae were established and validated in this study. The average amino-acid identity (AAI) cut-off value, 63.43 ± 0.01, was calculated to be an appropriate criterion for genus delineation of the family Desulfovibrionaceae. By applying the AAI cut-off value, 88 genomes of the Desulfovibrionaceae were divided into 27 genera, which follows the core gene phylogeny results. In this process, two novel genera (*Alkalidesulfovibrio* and *Salidesulfovibrio*) and one former invalid genus (“*Psychrodesulfovibrio*”) were officially proposed. Further, by applying the 95–96% average nucleotide identity (ANI) standard and the 70% digital DNA–DNA hybridization standard values for species delineation of strains that were classified as the same species, five strains have the potential to be newly classified. After verifying that the classification was appropriately performed through relative synonymous codon usage analysis, common characteristics were listed by group. In addition, by detecting metal resistance related genes *via in silico* analysis, it was confirmed that most strains display metal tolerance.

## Introduction

Sulfur is an essential element of biomolecules and an important factor in climate change through direct and indirect effects in the H_2_SO_4_ form, which brought attention to the sulfur cycle (Kellogg et al., 1972). The Desulfovibrionaceae family has been identified as one of the major contributor to the sulfur cycle on Earth. Discoveries of novel species are frequently reported as these species exist in various environments and can be easily cultured using well-established methods (Postgate, 1984; Widdel and Bak, 1992). Members of the family Desulfovibrionaceae have been employed as model organisms for sulfate reducing bacteria (SRB). Studies regarding anaerobic respiration have been actively conducted using *Desulfovibrio* since the 1950s, when dissimilatory sulfate reduction and a sulfate reductase named desulfoviridin (Postgate, 1956) were first identified in *Desulfovibrio* (Peck, 1959, 1961; Vosjan, 1975; Kobayashi et al., 1982; Barton et al., 1983; Lie et al., 1996; Matias et al., 2005; Pereira, 2008; Keller and Wall, 2011; Keller et al., 2014). Recently, interesting research results have been published on the effect of Desulfovibrionaceae family bacteria on host health. Although the exact correlation and mechanism have not been established, the results showed that the relative abundance of Desulfovibrionaceae was significantly increased in obese and metabolically impaired mice (Just et al., 2018), and the amount of *Desulfovibrio* spp. detected in the feces of Parkinson’s disease, patients showed a significant correlation with the severity of the disease (Murros et al., 2021). However, *Desulfovibrio* spp. do not always play negative roles. For example, *Desulfovibrio* spp. may live in sulfate depleted habitats in syntropy with methanogenic archaea (Scholten et al., 2007). SRB converts sulfate into sulfide, which reacts with heavy metals to form metal sulfide. Subsequently, spontaneous precipitation of metal sulfide achieves bioremediation (Ayangbenro et al., 2018). Aside from precipitation of sulfide, *Pseudodesulfovibrio hydrargyri* and *Pseudodesulfovibrio mercurii* are well-known mercury methylating bacteria, and activity against 10 ppb and 1 ppm inorganic Hg has been confirmed (Goñi-Urriza et al., 2020). *Desulfovibrio desulfuricans* showed resistance and high removal rates at concentrations of 50, 100, and 200 ppm in a mixed solution of cadmium nitrate tetrahydrate, nickel sulfate hexahydrate, and chromium oxide (Jeong et al., 2015). *Desulfovibrio magneticus* exhibited a sharp decrease in cadmium before the exponential phase when 1.3 ppm cadmium chloride was added to the medium (Arakaki et al., 2002). *Nitratidesulfovibrio vulgaris* was reported to tolerate a wide range of metal ions, such as 10 ppm Mn(II), 15 ppm Cr(III), 4 ppm Cu(II), 8.5 ppm Ni(II), and 20 ppm Zn(II) (Goulhen et al., 2006).

After the genus *Desulfovibrio* was first proposed in 1936 (Kluyver and Van Niel, 1936), the first attempt to classify this taxon, based on of DNA composition and physiological and biochemical properties, was crucial in establishing the classification criteria (Postgate and Campbell, 1966). In 2002, the genus *Desulfomonas* (Moore et al., 1976) was reclassified as a member of the genus *Desulfovibrio* based on molecular analysis (Loubinoux et al., 2002). Subsequently, the Desulfovibrionaceae family was officially recognized in 2006 (Kuever et al., 2005). The genera *Bilophila* (Baron et al., 1989), *Desulfovibrio* (Kluyver and Van Niel, 1936), and *Lawsonia* (McOrist et al., 1995) were reclassified into this family. Thereafter, *Desulfocurvus* (Klouche et al., 2009) and *Desulfobaculum* (Zhao et al., 2012) were added. The addition of several new genera, namely *Pseudodesulfovibrio* (Cao et al., 2016), *Halodesulfovibrio* (Shivani et al., 2017), *“Mailhella”* (Ndongo et al., 2017), *Desulfohalovibrio*, *Desulfocurvibacter* (Spring et al., 2019), “*Paradesulfovibrio”* (Kim et al., 2020), and *Desulfolutivibrio* (Thiel et al., 2020), finally led to a major reorganization based on the genome sequence data (Waite et al., 2020), resulting in Desulfovibrionaceae currently comprising nine validly published genera (*Fundidesulfovibrio, Humidesulfovibrio, Maridesulfovibrio, Megalodesulfovibrio, Nitratidesulfovibrio, Oleidesulfovibrio, Paradesulfovibrio, Paucidesulfovibrio*, and *Solidesulfovibrio*) and three published but not validated genera (*“Alteridesulfovibrio,” “Aminidesulfovibrio,”* and *“Frigididesulfovibrio”*) besides the existing genera. Reorganizing the confusing taxonomy of the Desulfovibrionaceae family was completed by adding two valid [*Macrodesulfovibrio* (Galushko and Kuever, 2020b), *Oceanidesulfovibrio* (Galushko and Kuever, 2020c)] and one invalid genera [“*Psychrodesulfovibrio*” (Galushko and Kuever, 2020d)] at the end of 2020 through Bergey’s manual (Galushko and Kuever, 2020a). As of August 2021, the Desulfovibrionaceae family comprised 21 validated genera, one synonym, and five non-validated genera.

The recently introduced classification method using the whole genome has been a powerful alternative to the two-step approach, which combined 16S rRNA gene sequence similarity and DNA–DNA hybridization (DDH) (Chun and Rainey, 2014). A genome sequence-based classification, such as average nucleotide identity (ANI) and digital DNA–DNA hybridization (*d*DDH), was introduced (Richter and Rosselló-Móra, 2009) to improve the resolution between highly similar interspecies sequences caused by the short length of the 16S rRNA sequence (Wambui et al., 2021). However, the resolution was insufficient for classification at the genus level, as it comprised only four nucleotide types. Therefore, as an alternative, the average amino acids identity (AAI)-based classification method was introduced to compare the genome information composed of amino acids (Konstantinidis and Tiedje, 2005b; Chun and Rainey, 2014; Rodriguez-R and Konstantinidis, 2014; Barco et al., 2020). There are several successful cases of reclassification using the whole genome, for example, dividing 39 strains belonging to 27 species of *Arcobacter* spp. into seven genera (Pérez-Cataluña et al., 2018) and reorganizing 91 genomes belonging to the existing three genera of the order *Methylococcales* into four genera (Orata et al., 2018). These cases followed a similar methodology, wherein the housekeeping genes were extracted, and phylogeny was performed using the ANI and *d*DDH values for the delineation of species, and percentage of conserved proteins (POCP) and AAI values for the delineation of genera. As the POCP is calculated using an amino acid sequence, it allows a higher resolution comparison for distant groups than the ANI or *d*DDH values that use a nucleotide sequence. Further, genome sequence-based reclassification of *Epsilonproteobacteria* and *Deltaproteobacteria*, to which SRB are affiliated, had been conducted (Waite et al., 2017, 2020). Although this method is not based on comparing genomic indices, it became the foundation for SRB classification by applying an alternative taxogenomic method based on phylogeny to a vast range of taxa and reorganizing them into a new phylum.

Although the results published in Waite et al. (2020) and Bergey’s manual (Galushko and Kuever, 2020a) almost corrected the confusing taxonomy of the family Desulfovibrionaceae, still issues require further investigation. First, the research did not provide a numerical value for genus delineation. Therefore, researchers proposing a new genus must perform extensive calculations and investigation. Comparing genomic indices becomes increasingly complex when studying large genera. Second, the classification was based solely on phylogeny results, indicating that the genus classification that introduced the numerical standard value comparison of genomic indices, which is considered a formal method of taxonomy, has not been made. Third, several mis-classified groups remain, particularly *Cupidesulfovibrio* [a genus newly proposed in early 2021 (Wan et al., 2021)] which requires further reclassification because it collides with the genus *Nitratidesulfovibrio*. Referring to the taxonomic comments on the Desulfovibrionaceae family in the 2020 version of Bergey’s manual (Galushko and Kuever, 2020a), some suggested that several species of *Pseudodesulfovibrio*, which are related to ‘*Paradesulfovibrio onnuriensis*’ IOR2^T^, deserve to be considered as an independent genus.

In this study, we performed phylogeny investigations using core genes extracted from all genomes belonging to Desulfovibrionaceae and roughly divided them into smaller groups. To evaluate the categorization process, several genomic indices, such as G + C content, *d*DDH, ANI, AAI, and POCP, were explored. Moreover, we not only arranged mis-classified strains at the species level but also proposed new genera reflecting phenotypes of other well-divided groups. This research will provide a genus cut-off genomic index that can be referenced without comparing extensive genome indices with previously reported species. In addition, the metal tolerance enzyme prediction using genome data highlights that the family Desulfovibrionaceae displays tolerance to certain extreme environments.

## Materials and Methods

### Bacterial Strains and Sequences

Information on all strains was collected from their related literature (Supplementary Table 1). All genomes of isolates, except for *Pseudodesulfovibrio tunisiensis* RB22^T^, registered under the Desulfovibrionaceae family were obtained from two public databases, the National Center for Biotechnology Information (NCBI) and EzBioCloud (Yoon et al., 2017a). *Pseudodesulfovibrio tunisiensis* RB22^T^ strains were ordered from JCM RICKEN and cultured for two days in DSM143 medium injected with H_2_/CO_2_ gas. Afterward, their genomic DNAs were extracted using QIAGEN DNeasy tissue kits and sequenced using the Oxford Nanopore PromethION sequencer according to NICEM’s commercial process. The raw data were *de novo* assembled using Flye v2.9-b1768 (Kolmogorov et al., 2019) and polished four times using Racon v1.5.0 (Vaser et al., 2017) and one time using Medaka v1.6.0.^1^ As a result, a complete genome of 1 contig of 3.6Mb size showing coverage of 675X depth was obtained (GenBank Assembly accession: GCA_022809775). To maintain the same conditions, all genomes were annotated using Prokka version 1.14.6 (Seemann, 2014). Information regarding CDS, rRNA, CRISPR repeat region, tRNA, and tmRNA was obtained from the Prokka output. Genome assembly statistics, such as number of contigs, bases (size), N50, and G + C content, were obtained using the Genome Assembly Annotation Service (GAAS) tool kit. Small subunit (SSU) rRNA sequences of the type strains were obtained from EzBioCloud. The data for strains with unpublished SSU rRNA sequences were directly exported from the annotation file for each strain.

### Phylogenetic Analyses

Core gene phylogeny was performed using PhyloPhlAn (Segata et al., 2013) with .faa result files obtained from Prokka. The phylogenomic tree was constructed with default options of 400 universal protein markers (Supplementary Table 2) and the following tools for the internal steps: USEARCH v5.2.32 (Edgar, 2010) for mapping into amino acid databases; MUSCLE v3.6 (Edgar, 2004) for multiple sequence alignment, and FastTree v2.1.10 (Price et al., 2009) with 1,000 bootstraps for phylogenic inference. The genomes of two strains belonging to the phylum *Rhodothermaeota* were downloaded from NCBI and used as an outgroup. In addition, the results were compared by phylogeny based on the 16S rRNA sequences. The 16S rRNA-based phylogenetic tree was multiple aligned using ClustalW (Chenna et al., 2003) in MEGA 6.0 (Tamura et al., 2013), and 1,000 bootstrap iterations were conducted to construct a tree using the neighbor-joining (Saitou and Nei, 1987) and the maximum-likelihood (White, 1982) methods with the Jukes–Cantor model (Jukes and Cantor, 1969) and the maximum-parsimony (Moore et al., 1973) method.

### Calculation of Genomic Indices

Average nucleotide identity values were calculated using the OAU (OrthoANI-usearch tool) (Yoon et al., 2017b), and *d*DDH (*digital*DDH, known as *is*DDH) was calculated using the Genome-to-Genome Distance Calculator (GGDC) (Meier-Kolthoff et al., 2013) provided by the DSMZ (Braunschweig, Germany). The AAI calculator by Kostas lab (Luo et al., 2014) was employed to compare two genomes written in amino acids with 20% minimum identity and 50 minimum alignments as alignment options. POCP is a comparison of the genomes as an amino acid sequence of two strains using BLASTP. The matched proteins with an E-value less than 10^–5^, a sequence identity over 40%, and a query cover of over 50% were regarded as conserved proteins. As strains belonging to the same genus share conserved proteins, with at least half of the whole protein, the strains showing a POCP value over 50% can be considered belonging to the same genus. POCP values were calculated as [(C_1_ + C_2_)/(T_1_ + T_2_)] × 100%, where C_1_ and C_1_ represent the conserved number of proteins in the two genomes, respectively; and T_1_ and T_1_ represent the total number of proteins in the two genomes being compared, respectively (Qin et al., 2014).

### Relative Synonymous Codon Usage Analysis

The open reading frame (ORF) search was performed as a priority to compare codon usage. The ORF finder function of the Sequence Manipulation Suite tool (Stothard, 2000) was used with the bacterial genetic code beginning with only ATG. The obtained ORFs were calculated using the bacterial genetic code for the codon usage function of the same tool. Analysis was conducted only for codons, excluding Met (ATG) and Trp (TGG), which are encoded by only one codon, and stop codons (TAG, TGA, and TAA) that encode no amino acids. A global test between codon bias in each group was conducted using Analysis of similarity (ANOSIM) with R’s vegan package (Oksanen et al., 2013; Gu et al., 2020). Principal component analysis (PCA) was performed using R’s prcomp formula, and since 93% of the variables could be explained with three principal components, 3D plotting was performed using plot_ly packages (Sievert, 2020). The distance between plots was obtained by introducing the coordinates into Equation 1.


(1) $$d={\left[{\left(X_{1}-X_{2}\right)}^{2}+{\left(Y_{1}-Y_{2}\right)}^{2}+{\left(Z_{1}-Z_{2}\right)}^{2}\right]}^{1/2}$$

### Comparison of Phenotype and Metal Resistance-Related Genes Between Groups

A literature study was conducted on all strains of the Desulfovibrionaceae family to investigate their phenotype. DIAMOND BLASTP was performed on the BacMet v2.0 database to identify metal resistance-related genes. Genes which satisfy the following conditions were searched: E-value lower than 10^–5^, 70% or more query coverage, and 30% similarity. The relative abundance of metal resistance-related genes was shown as a heatmap for each genome using ggplot2.

## Results

### Bacterial Strains and Genomes

All genomes (88 genomes) belonging to Desulfovibrionaceae were collected for subsequent analyses, and their basic information is summarized in Supplementary Table 1. A portion of the target genomes originate from host body isolates, including blood, gut, and feces (Moore et al., 1976; Baron et al., 1989; McOrist et al., 1995) but mainly from anaerobic environments such as sludge, mud, wastewater (sulfidic water), oil field, and marine sediment (Baena et al., 1998; Hernandez-Eugenia et al., 2000). Some members of this family have also been isolated from extreme environments, such as permafrost, ocean vent fields (hydrothermal chimneys), and heavy-metal-effected lake sediments (Ramsay et al., 2015; Ryzhmanova et al., 2019; Kim et al., 2020). Likewise, their genomic characteristics also showed large variations amongst isolation sources. Genome sizes ranged from 1.41 to 5.77 Mb, and the G + C content varied over a wide range (32.9–69.8 mol%). Genome statistics, such as contig number and N50, and characteristics, such as CDS, rRNA, tRNA, tmRNA, and CRISPR repeat regions, are summarized in Supplementary Table 1 along with the GenBank assembly accession number.

### Core Genes Phylogeny

**FIGURE 1:**
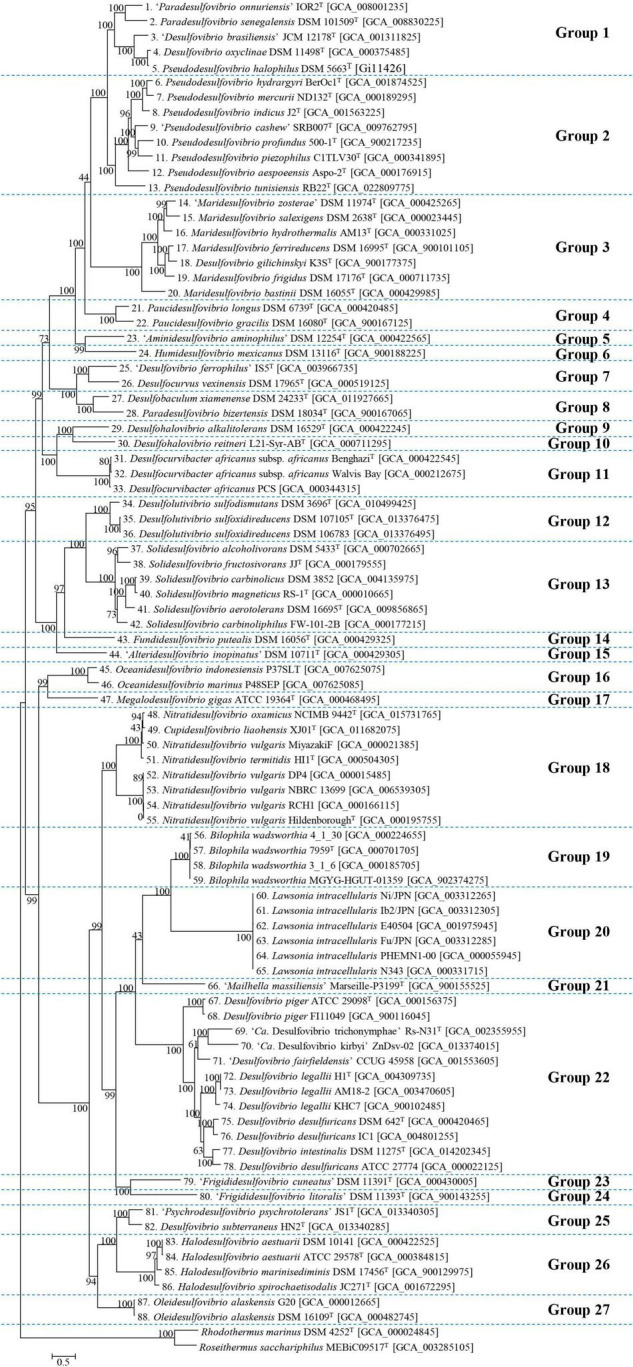
Phylogenomic tree of strains belonging to Desulfovibrionacae. Using 400 core gene markers, 88 Desulfovibrionaceae family strains were subdivided into 27 groups. Two type strains belonging to Rhodothermaceae were selected as outgroups. Each number before the scientific name implies the identical organism with the corresponding number at the subsequent analysis. The scale bar represents 5% estimated sequence divergence.

Phylogeny was performed using core genes extracted from previously collected genomes (Figure 1). A quick skim of a classified group with a difference of less than 1.5 distance, referred to the scale bar on the tree, suggested that 88 genomes, excluding the outgroup, could be subdivided into 27 groups. The results of clustering corresponded well with the validly published genera in most group cases. Although the genera constituting groups 5, 15, 21, and 23 are not officially recognized genera, each cluster resulting from those groups matched an individual genus. Group 1 is a mixture of the genera “*Paradesulfovibrio”* (“*Paradesulfovibrio onnuriensis*”), *Desulfovibrio* (“*Desulfovibrio brasiliensis”* and *Desulfovibrio oxyclinae*), and *Pseudodesulfovibrio* (*Pseudodesulfovibrio senegalensis*, *Pseudodesulfovibrio halophilus*). Group 8 is a mix of *Paradesulfovibrio* (*Paradesulfovibrio bizertensis*) and *Desulfobaculum* (*Desulfobaculum xiamenense*). In group 18, *Cupidesulfovibrio* and *Nitratidesulfovibrio* genera coexist. *Desulfohalovibrio alkalitolerans* belonging to group 9, has been classified as belonging to the same genus as *Desulfohalovibrio*, and with *Desulfohalovibrio reitneri* belong in group 10. However, as they show sufficient evolutionary distance, it was determined that they should be classified into separate groups. In the same vein, *Fdv*, from group 23, classified as “*Frigididesulfovibrio*” can also be proposed as a new genus. Last, group 18 is a cluster of two genera, *Nitratidesulfovibrio* (*Nitratidesulfovibrio oxamicus, Ntd. vulgaris*, and *Nitratidesulfovibrio termitidis*) and *Cupidesulfovibrio* (*Cupidesulfovibrio liaohensis*).

### Genome-Based Similarity Indices

Amino-acid identity and POCP are popular examples of genome-based similarity indices for genus delineation. The AAI cut-off value applied for the delineation of genera has a wide range of 60–80% (Konstantinidis and Tiedje, 2005a; Rodriguez-R and Konstantinidis, 2014). In previous studies on the Desulfovibrionaceae family, the AAI value of 60% was adopted to distinguish the genus by checking whether the existing genus satisfies these values (Spring et al., 2019). However, in reality, many taxa do not meet the AAI value criteria above, so it is necessary to verify whether this is an “appropriate AAI criterion that does not violate the monophyly rule” (Orata et al., 2018). Therefore, we devised a method to determine the threshold through clustering and scoring after much consideration. Subjective intervention was avoided by repetitive clustering and evaluation with gradual increment of cut-off parameters to establish unbiased criteria, and not setting strict borders after dividing the genus first. As the AAI cut-off value applied for each taxon varied, a Python script was written to objectively evaluate various thresholds and establish the criteria. This Python script automatically iterates clustering work and evaluates its result to determine an optimum threshold between 60.0 and 80.0. In the clustering step, threshold-based clustering was performed using the given pairwise AAI matrix (Supplementary Table 3). In the scoring step, inclusion of a member in multiple clusters was heavily penalized, whereas the single member group received relatively small point deductions. When the scoring is complete, the threshold is raised by 0.01 and the steps are repeated until the threshold reaches 80.00 (Supplementary Figure 1). For example, if there are three genomes A, B, and C, and A shows similarity above the given threshold with B and C, respectively, but B and C show similarity below the threshold with each other, Three clusters are created: [A, B, C], [A, B], and [A,C]. At this time, since A belongs to these three clusters, a large deduction is given. In order to distinguish the penalty given to the singleton member and the penalty given for overlapping clusters, the large deduction was given more than the total number of genome used. An executable file for Windows is available from the following repository: https://github.com/PMKYU98/monophyly_cutoff. Subsequently, the threshold that finally obtained the maximum score was set as the optimal AAI value. Therefore, there were five AAI threshold ranges calculated as possible thresholds (63.43 ± 0.01, 76.33 ± 0.23, 76.99 ± 0.17, 78.34 ± 0.11, and 78.50 ± 0.01). The threshold 63.43 ± 0.01 was determined to be the best, and when these values were set as cut-off values, it was confirmed that each genome belonged to one of the 27 independent groups without re-occurrence in multiple groups. These 27 groups matched the 27 groups abstracted in the previous phylogenomic tree. The second-best range, 76.33 ± 0.23, formed 49 groups, but an excessive number of unary groups occurred. Therefore, we selected 63.43 ± 0.01 as a promising maximum score, which is in the range of the recommended AAI threshold to classify the genus (Rodriguez-R and Konstantinidis, 2014), but slightly higher than the value used by Spring et al. (2019).

**FIGURE 2:**
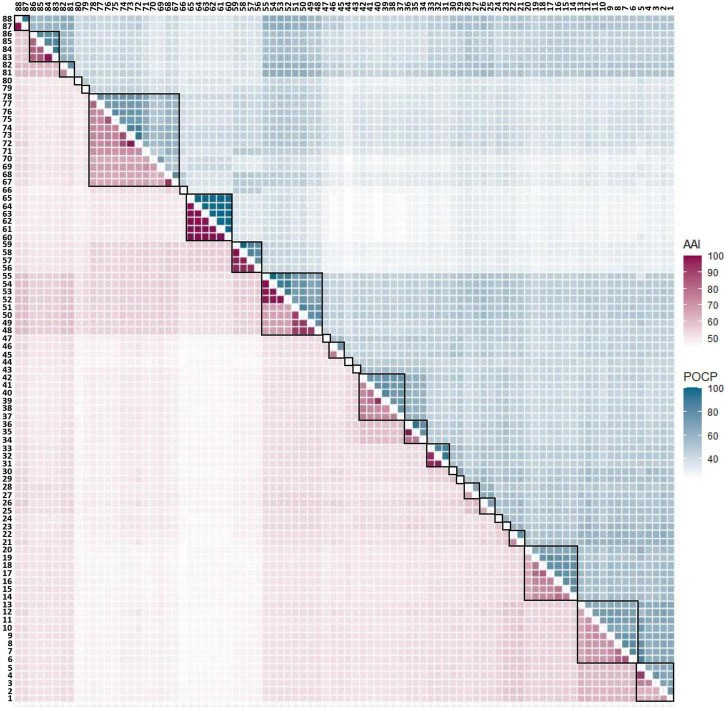
AAI and POCP from pairwise whole-genome comparisons. The values of AAI and POCP were expressed as heatmap. AAI is the lower left triangle indicated in purple color, and POCP is the upper right triangle indicated in cyan color. When the group was divided based on the AAI value alone, it was subdivided into 27 groups, which are separately surrounded by a black square on the figure.

POCP played the role of an auxiliary criterion besides the AAI value, and the value for genera delineation was set at 50% (Qin et al., 2014). However, when plotting the AAI and POCP values between genomes (Supplementary Figure 2), 50% POCP values did not correspond with the 63.43 ± 0.01 AAI cut-off values. Moreover, applying the 50% POCP cut-off violated the monophyly rule for taxon delineation. Previous studies have reported that the POCP cut-off cannot be an appropriate standard for genus delineation in several taxa (Aliyu et al., 2016; Li et al., 2017; Lopes-Santos et al., 2017; Orata et al., 2018; Wirth and Whitman, 2018). Therefore, it was decided not to adapt the POCP value as a criterion for genus delineation. The AAI and POCP results between each genome are displayed in a heatmap shown in Figure 2, and detailed values can be found in Supplementary Tables 3, 4.

Average nucleotide identity and *d*DDH are widely used for species delineation because their standard values are defined. If either ANI or *d*DDH values from the comparison of two genomes are under the cut-off of 95% ANI and 70% *d*DDH (Colston et al., 2014), they are considered as different species. Among the *Nitratidesulfovibrio vulgaris* strains belonging to group 18, the Miyazaki F strain should be classified as a different species. Group 22, including *Desulfovibrio piger* FI11049, *Desulfovibrio legallii* KHC7, *Dsv. desulfuricans* IC1 and *Dsv. desulfuricans* ATCC 27774, should be separated into new species from their type strains. Excluding the strains mentioned above, every other strain met both the ANI and *d*DDH criteria, and therefore, did not require additional classification. The ANI and *d*DDH results between each genome are summarized in a heatmap and shown in Supplementary Figure 3. Detailed values are provided in Supplementary Tables 5, 6.

### Comparison for Codon Bias

**FIGURE 3:**
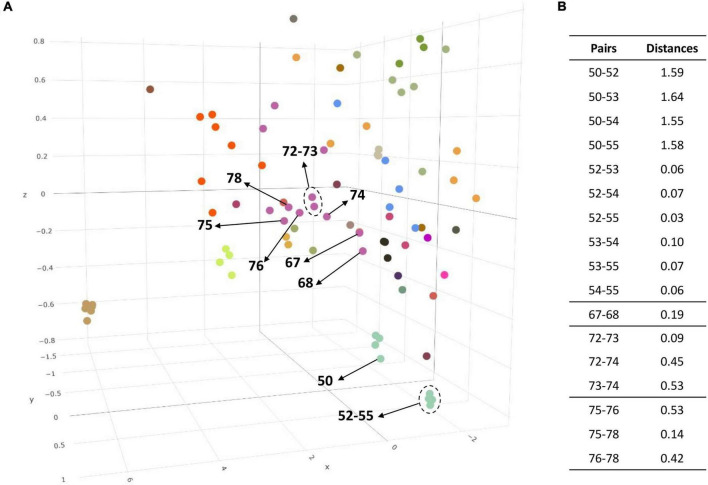
**(A)** 3D plot of the three major axes generated by principal component analysis (PCA) of the RSCU values. The RSCU of each strain except for the 5 codons of Met, Trp, and Stop codons were plotted after PCA analysis. In this scatter plot, the same color indicates the same genus and the label of each dot can be found in the accompanying.html file. **(B)** The distance on the PCA plot between the existing group and the strain to be reported as a novel species. 50, *Ntd. vulgaris* Miyazaki F; 52, *Ntd. vulgaris* DP4; 53, *Ntd. vulgaris* NBRC 13699; 54, *Ntd. vulgaris* RCH1; 55, *Ntd. vulgaris* Hildenborough^T^; 67, *Dsv. piger* ATCC 29098^T^; 68, *Dsv. piger* FI11049; 72, *Dsv. legallii* H1^T^; 73, *Dsv. legallii* AM18-2; 74, *Dsv. legallii* KHC7; 75, *Dsv. desulfuricans* DSM 642^T^; 76, *Dsv. desulfuricans* IC1; 78, *Dsv. desulfuricans* ATCC 27774.

Codon usage can reflect evolutionary processes because it is influenced by the G + C content, replication strand skew, or gene expression (Sharp et al., 2005). Thus, we calculated the relative synonymous codon usage (RSCU) values and measured codon usage bias for each group to verify their evolutionary relationship and clustering (Supplementary Table 7). The RSCU values obtained for each genome were then calculated and plotted. (Figure 3A and Supplementary Figure 4). ANOSIM was used to observe the similarity in the codon usage bias of each group. The R statistic was 0.678 and the *p*-value was 0.0001 in the global test result. These figures indicate each genus group has a codon usage bias that differs significantly from other groups, suggesting that the group previously divided at the genus level was sufficiently discriminated. In addition, the PCA using the RSCU values also verified whether they were the same species. Figure 3 shows the distance between them more intuitively in the 3D plot. The numbers in parentheses next to the scientific name in the following statements indicate the label in the figure. Four strains of *Ntd. vulgaris*, DP4, NBRC 13699, RCH1 and Hildenborough^T^ (52–55), were plotted closely (distance: 0.065 ± 0.035), whereas the other *Nitratidesulfovibrio vulgaris* strain, Miyazaki F (50), was plotted slightly further away (distance:1.59 ± 0.04). Similarly, *Dsv. piger* FI11049 (68), *Dsv. legallii* KHC7 (74), *Dsv. desulfuricans* IC1 (76), and *Dsv. desulfuricans* ATCC 27774 (78), which were separated into new species, showed sufficient distance to be separated from strains belonging to the existing group (Figure 3B).

### Phylogenetic Analysis Based on 16S rRNA Sequence

**FIGURE 4:**
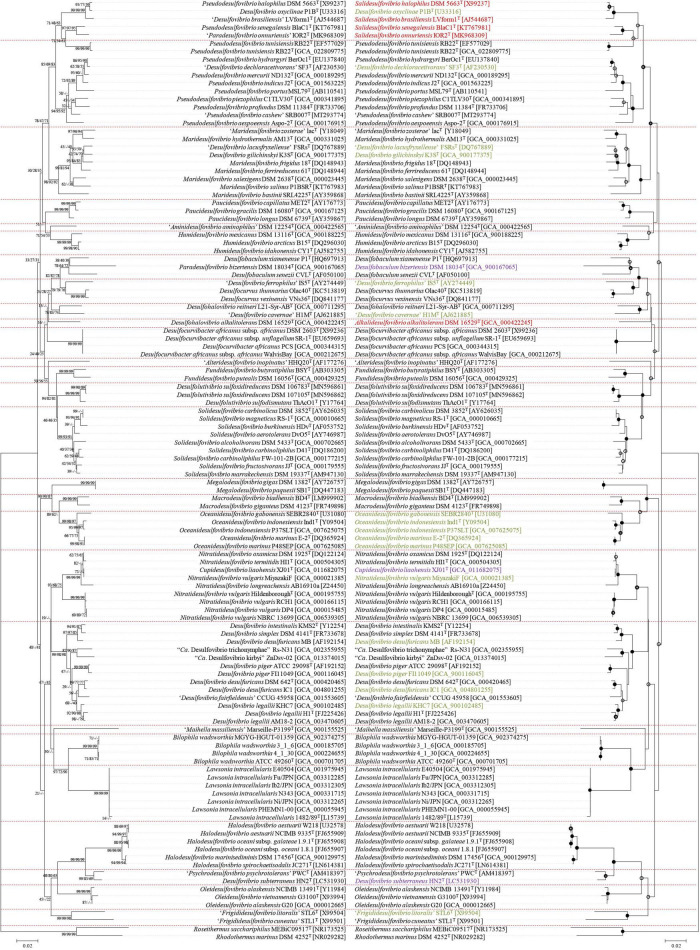
Reclassification of the Desulfovibrionaceae by 16S rRNA gene sequence similarity inferred by genomic tree. This figure includes mirror image. Left sided tree is before reclassifications, Right sided tree is after reclassifications. Purple colored label, rearrangement through this study; Red colored label, proposed as a new genus; Green colored label, which has the potential to be newly classified, but requires further researches because it cannot be achived at this moment: lack of detailed phenotypic characterization or lack of available genome sequence or lack of available culture in two public culture collections. The tree is based on the Jukes & Cantor distances model and the neighbor-joining method with 1,000 bootstraps. Nodes with branch support > = 70% recovered by the three algorithms (the neighbor joining, the maximum-likelihood, and the maximum-parsimony algorithm) were indicated with •; Nodes recovered by the three methods but with < 70% bootstrap values were indicated with ○°  ; Nodes recovered by two of the above methods were indicated with °.

Based on the previous analysis, 88 genomes were divided into 27 genera. During the process, two new genera were added and the possibility of six new species was confirmed. However, since there are many species in the Desulfovibrionaceae family without identified genome sequences, extended phylogenetic analysis using 16S rRNA sequence had to be performed. For this, type strains corresponding to all species and subspecies of taxa registered in the Ez-taxon DB were listed together. To compare with the previous core gene phylogenetic tree, 16S rRNA sequences of strains used for genome analysis were also added. With strains not registered with the 16S rRNA sequence, the sequence was directly extracted from the annotation results. The results are summarized in Figure 4 (illustrated as a mirror image; the left side indicates previously published taxa names, and the right side indicates newly reorganized taxa names), which implies that most of the type strains belonging to Desulfovibrionaceae are well grouped according to new classification groups, as determined by our analysis. *Psd. tunisiensis* RB22^T^ belonged to the *Pseudodesulfovibrio* group in the phylogenetic analysis using the genome sequence. Still, it was classified to the *Salidesulfovibrio* group in analysis using the 16S rRNA sequence. *Dsv. cavernae* H1M^T^, which was grouped with *Dhv. reitneri* L21-Syr-AB^T^, appears to be far enough to be classified as a different genus. However, as reclassification using the genome sequence was impossible, the status of this species remains to be investigated.

### Defining Phenotype and Genotype Characteristics for Each Group

To define the characteristics of the newly classified groups, a literature search was conducted on the characteristics of the phenotype for each strain. The information obtained is summarized in Table 1 by group (genus). Most show morphology in the form of curved rods (vibrio) and have motility with a single polar flagellum. *Paucidesulfovibrio* and *Desulfovibrio* include members of the spirillum type and *Desulfohalovibrio*, *Oceanidesulfovibrio*, and *Nitratidesulfovibrio* include sigmoid types, inferring that one genus can show various morphologies, as well as vibrio. Regarding growth conditions, the characteristics of each group were more pronounced. Particularly, depending on whether the taxa comprised terrestrial-derived species or marine-derived species, they either did not require salt or demanded a NaCl concentration of 2% or more for optimal growth. In addition, the newly classified genera, *Alkalidesulfovibrio*, require an optimal pH of higher than 8, which is higher than that required by other genera (generally pH 7). Regarding the G + C content, the difference between the maximum and minimum values in each group of *Frigididesulfovibrio*, *Pseudodesulfovibrio, Desulfocurvus, Desulfobaculum*, and *Desulfovibrio* were 16.6, 13.9, 13.7, 11.3, and 10.6, respectively, showing a difference of over 10 mol%, whereas in other groups, the gap was only around 5 mol%.

**TABLE 1:** Phenotype and genotype information of the genera of the family Desulfovibrionaceae.

Genus		*Alkalidesulfovibrio*	*“Alteridesulfovibrio”*	*“Aminidesulfovibrio”*	*Bilophila*	*Desulfobaculum*	*Desulfocurvibacter*	*Desulfocurvus*	*Desulfohalovibrio*	*Desulfolutivibrio*
Morphology		Vibrio	Vibrio	Vibrio	Pleomorphic rod with swollen ends	v (Rod or Vibrio)	Vibrio	v (Rod or Vibrio)	v (Sigmoid or Vibrio)	Vibrio
Flagellation		Single polar	Single polar	Single polar	–	Single polar	Lophotrichous	Single polar	Single polar or Monopolar bitrichous	Single polar
Motility		+	+	+	–	+	+	+	+	+
G + C content (genome)		64.5	49.1	66.2	59.2–59.3	52.3–63.6	61.1–61.4	56.0–69.7	65.5	63.5–64.1
Respiration Quinone		MK-7	Nr	Nr	Nr	MK-7^a^	MK-7^b^	Nr	MK-7/MK-6^c^	MK-7
pH range (Opt.)		6.9–9.9 (9.0–9.4)	5.0–8.0 (6.9–7.2)	6.7–8.0 (7.5)	Nr	6.0–8.1 (7.0–7.6)	6.3–7.7 (7.0)	Nr (6.9–7.1)	5.5–8.5 (7.5)	6.5–8.5 (6.5–7.3)
Temp. range (Opt.) ^o^C		16–47 (43)	15–42 (30)	25–40 (35)	Nr (35–37)	15–42 (35–40)	20–40 (37)	Nr (37–40)	20–48 (37–40)	15–45 (35–37)
Salinity. range (Opt.)%		0.085–0.7 (0.13)	0.5–2.0 (1.0)	0–2.0 (0.05–0.75)	Nr	0–12.5 (0.5–2.5)	0–4.25 (0–1.0)	0–5.0 (0.2)	0.7–18.0 (4.0–6.0)	0.1–2.0 (0.1–0.7)
Fermentative growth		+	–	+	Nr	v+	–	+	+	Nr
Major electron donors	All	H_2_†, Formate†, Lactate, Pyruvate	H_2_‡, Formate‡, Ethanol, Lactate, Pyruvate, Fumarate, Malate, Sugars	Formate‡, Ethanol‡, Lactate, Pyruvate, Amino acids	Pyruvate	H_2_†, Lactate, Pyruvate	H_2_†, Formate†, Ethanol, Lactate, Pyruvate	Formate‡, Lactate, Pyruvate	Formate†, Lactate, Pyruvate, Ethanol	H_2_‡, Ethanol, Lactate
	Most					Fumarate, Succinate, Malate, Cysteine		H_2_†		
	Some					C2–C4 alcohols		Iron	H_2_†, C1 and C3-C5 alcohols, Fumarate, Succinate, Malate, Glycerol	Formate‡
Major electron acceptors	All	Sulfate, Sulfite, Thiosulfate	Sulfate, Sulfite, Thiosulfate	Sulfate, Sulfite, Thiosulfate	Nitrate	Sulfate, Sulfite	Sulfate, Sulfite, Thiosulfate, Sulfur(w)	Sulfate, Sulfite, Thiosulfate	Sulfate, Sulfite, Thiosulfate	Sulfate, Sulfite, Thiosulfate
	Most					Thiosulfate, Fumarate				
	Some					Sulfur			Nitrate	DMSO
Isolation source		Metal coupon in corroison monitoring reactor in District heating plant	Marine sediment	Wastewater	Intra-abdominal specimen	Marine sediment, solar saltern	Marine sediment, well water	Deep ground water, marine sediment, wastewater treatment reactor	Microbial mat of hypersaline lake, deep subsurface	Sewage sludge, freshwater mud
References		Abildgaard et al., 2006	Reichenbecher and Schink, 1997	Baena et al., 1998	Baron et al., 1989	Haouari et al., 2006; Zhao et al., 2012; Tsu et al., 1998	Brown et al., 2011; Campbell et al., 1966; Spring et al., 2019; Castañeda-Carrión et al., 2010; Brown et al., 2011	Klouche et al., 2009; Hamdi et al., 2013; Dinh et al., 2004	Spring et al., 2019; Sass and Cypionka, 2004	Thiel et al., 2020; Bak and Pfennig, 1987

**Genus**		***Desulfovibrio***	***“Frigididesulfovibrio”***	***Fundidesulfovibrio***	***Halodesulfovibrio***	***Humidesulfovibrio***	***Lawsonia***	***Macrodesulfovibrio***	***“Mailhella”***	***Maridesulfovibrio***

Morphology		v (Rod or sprillum or **Vibrio**)	Vibrio	Vibrio	Vibrio	Vibrio	Vibrio	v (Rod or vibrio)	Rod	v (Rod or **Vibrio**)
Flagellation		Single polar	Single polar	Single polar	Single polar	Single polar	–	Single polar	Nr	**Single polar** or Monopolar bitrichous
Motility		v+	+	+	+	v+	–	+	–	+
G + C content (genome)		53.6–64.2	36.9–53.5	62.8	45.0–46.2	65.0–65.6	32.9–33.1	54.6–59.5^∨^	59.1	41.8–47.1
Respiration Quinone		MK-6^d^	Nr	MK-6(H_2_)^e^	MK-6^f^, MK-6(H2)^g^	MK-6(H2)	Nr	Nr	Nr	MK-6(H2)^h^
pH range (Opt.)		4.9–9.0 (7.0–7.5)	Nr	6.2–8.0 (7.0–7.1)	6.5–8.5 (7.0–8.0)	6.0–8.2 (6.5–7.2)	Nr	5.5–8.5 (7.5–7.9)	Nr	5.2–8.5 (5.8–7.8)
Temp. range (Opt.) ^o^C		10–45 (28–37)	0–33 (28)	20–40 (30–35)	10–40 (20–37)	2–40 (24–37)	Nr (35–37)	15–45 (35–37)	Nr	0–50 (19–40)
Salinity. range (Opt.)%		0–3.0 (0–0.5)	0.25 (Nr)	0–6.0 (0–1.0)	0.05–6.0 (2.0–3.5)	0–2.0 (0–0.2)	Nr	0.2–8.0 (0.2–3.0)	Nr	0–12.0 (1.0–4.0)
Fermentative growth		v+	+	+	+	v+	Nr	+	Nr	v+
Major electron donors	All	H_2_‡, Ethanol, Lactate, Pyruvate	H_2_‡, Formate‡, Lactate, Pyruvate, Fumarate	H_2_†, Ethanol, C4 alcohols, Lactate, Pyruvate	Formate‡, Lactate, Pyruvate, Fumarate, Malate	H_2_†, Formate†, Lactate, Pyruvate	Nr	H_2_‡ Lactate, Pyruvate	Nr	Formate†, Lactate, Pyruvate,
	Most	Fumarate, Succinate			H_2_‡, Glycerol, Succinate	Ethanol				H_2_†, Ethanol, Fumarate
	Some	Formate‡, C3-C4 alcohols, Malate, Aromatic aldehydes, Alanine	Ethanol, Malate	C3 alcohols, C4–C5 fatty acids, Fumarate, Malate, Butyrate	Ethanol, C3 alcohols, Amino acids, Sugars	Fumarate, Succinate, Malate, Amino acids,		Formate‡, Ethanol, C3–C4 alcohols, Glycerol, Fumarate, Cysteine		Glycerol, C3–C4 alcohols, Succinate, Malate†, Amino acids, Sugars
Major electron acceptors	All	Sulfate, Thiosulfate	Sulfate, Sulfite, Thiosulfate, Sulfur	Sulfate, Thiosulfate	Sulfate, Sulfite, Thiosulfate	Sulfate, Sulfite, Thiosulfate, Sulfur, DMSO	Nr	Sulfate, Sulfite, Thiosulfate	Sulfate	Sulfate, Sulfite, Thiosulfate
	Most	Sulfite			Taurine, Fumarate					
	Some	Sulfur, Nitrate, Fumarate, Metal ions^#^		Sulfite, Fumarate		Fumarate		Sulfur		Sulfur, Fumarate
Isolation source		Hindgut of termite, rumen of a sheep, human faeces and blood, tar and sand mix, anaerobic sludge	Littoral sediment	Well water, sewage sludge	Anoxic sea sediment, sea water, soil	Anaerobic sludge of cheese wastewater, permafrost, sediment	Intestines of animals, intracellular parasite	Thermal spring, lagoon sediment	Human faeces	Saline and freshwater lake, sediment, hydrothermal chimney, seagrass, permafrost, deep subsurface oil well
References		Loubinoux et al., 2002; Zellner et al., 1989; Jyothsna et al., 2008; Thabet et al., 2010	Sass et al., 1998	Basso et al., 2005; Suzuki et al., 2010	Stüven, 1960; Postgate and Campbell, 1966; Finster and Kjeldsen, 2010; Shivani et al., 2017; Takii et al., 2008	Hernandez-Eugenia et al., 2000; Pecheritsyna et al., 2012; Sass et al., 2009	McOrist et al., 1995	Fadhlaoui et al., 2015; Esnault et al., 1988	Ndongo et al., 2017	Magot et al., 2004; Nielsen et al., 1999; Alazard et al., 2003; Postgate and Campbell, 1966; Ben Ali Gam et al., 2018; Ryzhmanova et al., 2019; Vandieken et al., 2006; Sattley and Madigan, 2010

**Genus**		***Megalodesulfovibrio***	***Nitratidesulfovibrio***	***Oceanidesulfovibrio***	***Oleidesulfovibrio***	***Paucidesulfovibrio***	***Pseudodesulfovibrio***	***Psychrodesulfovibrio***	***Salidesulfovibrio***	***Solidesulfovibrio***

Morphology		Vibrio	v (Sigmoid or **Vibrio**)	v (Sigmoid or **Vibrio**)	Vibrio	v (Rod or vibrio or spirillum)	Vibrio	Vibrio	v (Rod or **Vibrio**)	v (Rod or **Vibrio**)
Flagellation		Single polar	**Single polar** or Monotrichous polar	Single polar	Single polar	Single polar	Single polar or peritrichous	Single polar	v (Negative or **Single polar**)	Single polar
Motility		+	+	+	+	+	+	+	+	v+
G + C content (genome)		63.0–63.3	63.2–67.1	60.4–62.4	57.8–64.1	58.4–63.6	49.9–65.2	56.7–59.3	58.1–61.0	61.6–66.5
Respiration Quinone		MK-6^i^	Nr	Nr	Nr	Nr	MK-6(H2)^j^	MK-6(H2)^k^	Nr	MK-6^l^, MK-7(H2)^m^
pH range (Opt.)		6.5–8.5 (7.2–7.4)	5.5–9.0 (6.6–7.4)	6.4–8.5 (6.9–7.3)	5.0–10.0 (7.0–7.5)	5.4–8.8 (6.8–7.4)	4.5–9.9 (6.5–7.5)	6.0–10.0 (7.2–9.0)	4.5–9.0 (6.5–7.6)	5.3–8.7 (6.5–7.3)
Temp. range (Opt.) ^o^C		10–45 (30–36)	16–50 (35–37)	10–50 (30–37)	10–45 (37)	10–50 (35–40)	4–65 (25–35)	10–50 (28–35)	15–45 (30–40)	3–50 (29–38)
Salinity. range (Opt.)%		0–10 (0)	0–5.0 (0–0.1)	0–17.0 (5.0–6.0)	0–10.0 (2.5–5.0)	0–12.0 (1.0–6.0)	0–10.0 (0.6–8.0)	0–4.0 (0–0.5)	0.5–22.5 (3.0–10.0)	0–5.0 (0–1.0)
Fermentative growth		+	+	+	v	v–	+	+	v+	+
Major electron donors	All	H_2_†, Formate†, Lactate, Pyruvate, Fumarate, Succinate, Malate	Lactate, Pyruvate	H_2_‡, Formate‡, Ethanol, Lactate, Pyruvate, Fumarate, Succinate, Malate, Fructose	Formate‡, Ethanol, Glycerol, Lactate, Pyruvate, Fumarate, Malate	H_2_‡, Formate‡, Lactate, Pyruvate	Lactate	Ethanol, Lactate, Pyruvate, Succinate, Malate	Lactate, Pyruvate	Ethanol, Lactate, Pyruvate, Fumarate
	Most		H_2_†, Formate†, Ethanol, Fumarate, Malate, Organic acids				H_2_†, Pyruvate, Fumarate, Malate		H_2_†, Formate†	H_2_‡, Formate‡, Glycerol, C3–C5 alcohols, Malate
	Some	Ethanol, C3–C5 alcohols, Glycerol	C1 and C4 alcohols, Sugars	C4 alcohols	Succinate, Ethanol, C4 alcohols		Formate†, Fatty acids^∧^, Ethanol^∧^, C1 and C3–C4 alcohols, Succinate, Amino acid	H_2_†, Formate†, C4 alcohols, Fumarate, Cysteine	Ethanol, Glycerol, C1 and C3–C4 alcohols, Fumarate, Succinate, Malate, Amino acids	C1 alcohols‡, Succinate
Major electron acceptors	All	Sulfate, Sulfite, Thiosulfate, Sulfur	Sulfate, Sulfite	Sulfate, Sulfite, Thiosulfate, Sulfur	Sulfate, Sulfite Thiosulfate	Sulfate, Sulfite, Thiosulfate, Sulfur	Sulfate, Sulfite, Thiosulfate	Sulfate	Sulfate, Sulfite, Thiosulfate	Sulfate, Thiosulfate
	Most		Thiosulfate			Fumarate			Sulfur	Sulfite, Sulfur
	Some	Fumarate, Haloaromatic compounds	Sulfur, Iron(III), Nitrate, Nitrite, Oxygen, Fumarate	Fumarate	Fumarate		Sulfur, Fe(III), Nitrate, Nitrite, DMSO, Fumarate	Sulfite, Thiosulfate, Fe(III), Mn(IV), DMSO, Fumarate, AQDS	Fe(III), DMSO, Oxygen, Fumarate	Fumarate, Malate
Isolation source		Water, wastewater from a zinc smelter	Heavy metal impacted sediment, uranium mining waste piles, hindgut of a termite, degraded paddy field, oilfield fluids, chicken feed	Sea water from oilfield, water from oil pipeline	Oil field	Oil field	Marine sediment, deep subsurface, ground water	Mud and ice, deep sea surface sediment	Marine sediment, microbial mat in saline lake, oil refinery plant wastewater, dolomite	Waste water, sulfide-rich sediment, ricefield soil, contaminated ground water
References		Le Gall and Dragoni, 1966; Morais-Silva et al., 2014; Van Houten et al., 2009	Trinkerl et al., 1990; Aketagawa et al., 1985; Yagi, 1969; Ogata and Yagi, 1986; Ozawa et al., 2000; López-Cortés et al., 2006; Wan et al., 2021; Redburn and Patel, 1994; Voordouw, 2002; Heidelberg et al., 2004	Feio et al., 1998; Thabet et al., 2007; Tardy-Jacquenod et al., 1996	Dang et al., 1996; Feio et al., 2004	Magot et al., 2004; Miranda-Tello et al., 2003; Magot et al., 1992	Ranchou-Peyruse et al., 2018; Sun et al., 2000; Cao et al., 2016; Suzuki et al., 2010; Motamedi and Pedersen, 1998; Bale et al., 1997; Khelaifia et al., 2011 Ben Ali Gam et al., 2009	Jyothsna et al., 2008	Kim et al., 2020; Thioye et al., 2017; Warthmann et al., 2005; Krekeler et al., 1997; Caumette et al., 1991	Nanninga and Gottschal, 1987; Sakaguchi et al., 2002; Ouattara et al., 1999; Mogensen et al., 2005; Ramsay et al., 2015; Allen et al., 2008; Qatibi et al., 1991; Vainshtein et al., 1992; Ollivier et al., 1988; Chamkh et al., 2009

*†: Require acetate or yeast extract.*

*Nr: Not reported.*

*‡: Require acetate.*

*Bold type: predominant characteristic.*

*^∧^: Require 2-chlorophenol.*

*v: variable.*

*∨: G + C content measured based on HPLC.*

*v+: variable but positive is predominant.*

*#: only in sulfate-free conditions.*

*v-: variable but negative is predominant.*

*+ : positive.*

*(w): weak activity.*

*-: negative.*

*a: Desulfobaculum xiamenense.*

*b: Desulfocurvibacter africanus benghazi.*

*c: Desulfohalovibrio reitneri.*

*d: Desulfovibrio piger, Desulfovibrio legallii.*

*e: Fundidesulfovibrio butyratiphilus.*

*f: Halodesulfovibrio aestuarii, Halodesulfovibrio oceani, Halodesulfovibrio spirochaetisodalis.*

*g: Halodesulfovibrio marinisediminis.*

*h: Maridiesulfovibrio gilichinskyi.*

*i: Megalodesulfovibrio gigas.*

*j: Pseudodesulfovibrio portus.*

*k: Psychrodesulfovibrio subterraneus.*

*l: Solidesulfovibrio alcoholivorans, Solidesulfovibrio fructosivorans.*

*m: Solidesulfovibrio magneticus.*

### Comparison of Metal Resistance-Related Gene Abundance Between Groups

**FIGURE 5:**
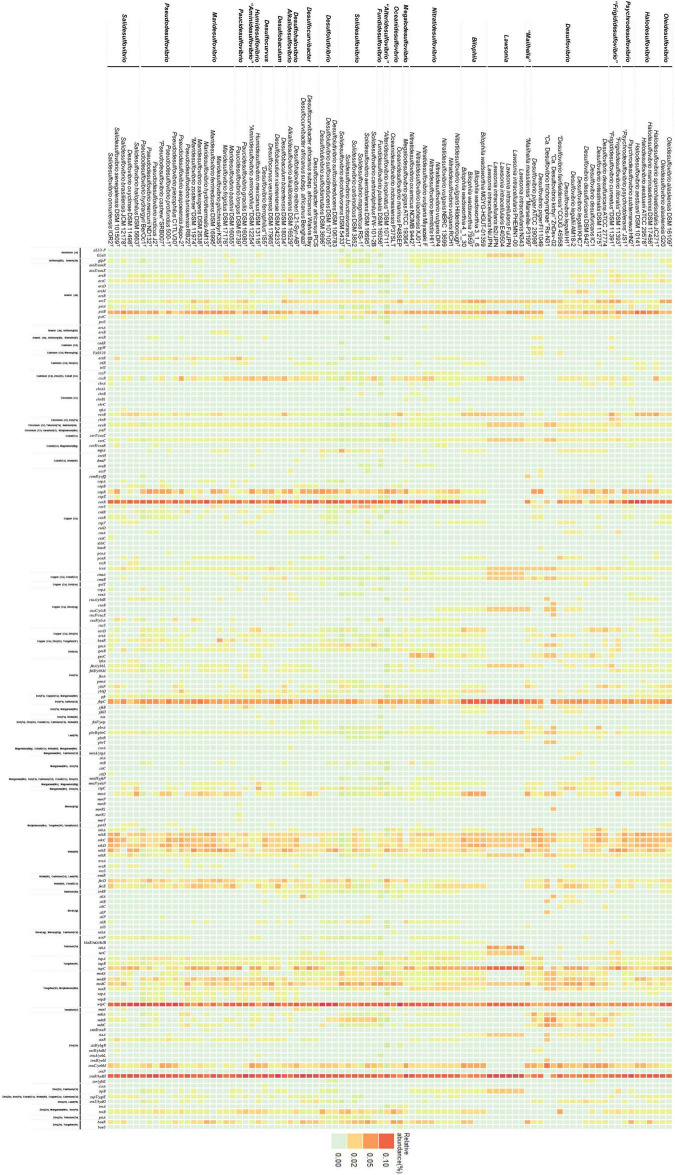
Heatmap of metal related genes. This figure verifies that there is a distinguishable difference in pattern of metal resistance related genes between each group.

Most of the Desulfovibrionaceae are isolated from habitats with high metal concentrations (Supplementary Table 1). This inspires interest in the search for metal resistance genes in the Desulfovibrionaceae family. Most species belonging to the family exhibit metal resistance by forming metal-sulfide precipitates, resulting from sulfate reduction. Besides forming metal-sulfide precipitates, several mechanisms are required for metal resistance, including import system regulation, efflux system, extracellular barrier, and reduction. Because many previous studies explored the genes related to sulfate reduction in Desulfovibrionaceae taxa, we focused on other metal resistance genes. Through this analysis, we aimed to explore species with the potential to show metal resistance, and to determine differences between species showing resistance potential and species that do not. The result of PCA of relative abundance data indicates significantly different patterns between each group (Supplementary Figure 5). Although not all species were investigated, obvious trends can be observed within the data. While most Desulfovibrionaceae family members display many metal resistance-related genes (Figure 5), several strains of the genus *Desulfovibrio*, *Lawsonia*, *Bilophila*, and “*Mailhella*” have low level gene abundances of metal import system regulators, such as *copR* and *corR*. They were commonly isolated from the biotic environment (intestine, feces, and blood). Considering that other strains were isolated from environments where metal elements are easily accessible (hydrothermal vent field, heavy metal affected sediment, aquifer, and mud), we estimate that the environmental condition of the habitat is reflected. Conversely, groups under the monophyly are adjacent in a PCA 3D plot (Supplementary Figure 5), which implies that gene abundance patterns of evolutionarily related species show a relative resemblance. Although the analysis was limited to species with identified genome sequences, we observed a distinction between each genus. Regarding *Solidesulfovibrio* and *Desulfovibrio*, the abundance of the multidrug efflux system is greater than other genera. The gene abundance related to arsenate methylation was relatively high in the genera *Solidesulfovibrio*, *Pseudodesulfovibrio*, *Desulfolutivibrio*, and *Desulfovibrio*. The mercury reductase gene, *merA*, displayed great abundance in the genera *Maridesulfovibrio*, *Desulfocurvibacter*, “*Mailhella*”, *Desulfovibrio*, and *Bilophila* (refer to Supplementary Table 8 for details).

## Discussion and Conclusion

The literature shows several gaps in knowledge regarding the current taxonomy of the family Desulfovibrionaceae. Therefore, this study reclassified the mis-classified taxa. In this process, the entire species was reclassified and expanded to the type strain of the species without genome sequencing. According to previous analysis, a significant number of taxa require classification. However, to propose a new taxon or to change an existing taxon, Rule 30-3b of the International Code of Nomenclature of Prokaryotes (ICNP) must be followed (Parker et al., 2019), it indicates that the type strain must be deposited into two publicly recognized public culture collections. The classification cannot be ideally completed because some taxa of Desulfovibrionaceae do not meet the mentioned condition due to deposits remaining in only one culture collection or loss of resources (*Dsv. legallii* KHC7, *Dsv. desulfuricans* IC1, *Dsv. piger* FI11049, *Dsv. litorals* DSM 11393, “*Dsv. cavernae*” H1M, “*Dsv. ferrophilus*” IS5, “*Dsv. lacusfryxellense*” FSRs, “*Dsv. dechloracetivorans*” SF3, “*Dsv. oxyclinae*” P1B), while some others lack the complete phenotypic description and chemotaxonomic characterization required to be proposed as a new species (*Dsv. desulfuricans* ATCC 27774 and *Ntd. vulgaris* Miyazaki F), which are the common limitations shared with previous research (Galushko and Kuever, 2020a; Waite et al., 2020). Despite the unclear distinction between *Oceanidesulfovibrio* and *Macrodesulfovibrio* in phylogeny based on the 16S rRNA sequence, this study with AAI-based criteria could not confirm that *Oceanidesulfovibrio* and *Macrodesulfovibrio* should be reorganized into the same genera (Figure 1, 4) due to the lack of reported genomes of strains belonging to those groups. Therefore, it is necessary to conduct a classification study applying the AAI classification standard value suggested when the genome of any type strain belonging to *Macrodesulfovibrio* is reported. In the case of *Pseudodesulfovibrio tunisiensis*, 16S rRNA phylogeny showed that the bacteria clearly belong to the *Salidesulfovibrio* group, but genome phylogeny showed that it belongs to the *Pseudodesulfovibrio* group. *Marixanthomonas spongiae* HN-E44^T^ has an AAI value of 85.2% with *Marixanthomonas ophiurae* JCM 14121^T^. Similarly, AAI and 16S rRNA sequence similarity analysis results conflict also in the case of *Marixanthomonas spongiae* HN-E44^T^ and *Marixanthomonas ophiurae* JCM 14121^T^. Two bacteria have an AAI value of 85.2%, and this exceeds the AAI criteria for genus delineation of Flavobacteriaceae. However, they showed only a 16S rRNA sequence similarity of 93.6%, which is lower than the minimum identity value of 94.9 ± 0.4% to guarantee the circumscription of a single genus (Yarza et al., 2008). This inconsistency is unaccountable yet, but several possibilities can be discussed. An introduction of an external 16S rRNA sequence might be charge of this discrepancy. However, the three 16S rRNA sequences from *Pseudodesulfovibrio tunisiensis* were highly similar, so this possibility should be excluded. Another possibility is that the evolutionary rate of the rRNA sequence and the genome did not match. It can happen when a large amount of external functional genes are introduced into the genome through various mechanisms, including horizontal gene transfer. This hypothesis is supported by the vast G + C content range of the *Pseudodesulfovibrio* group from 49.9 to 65.2.

Finally, two novel genera were proposed and the possibility of five independent species was confirmed. In addition, nine species were reclassified into different genera. According to rule 23a of the ICNP, “Each taxon above species, up to and including order, with a given circumscription, position, and rank can bear only one correct name, that is, the earliest that is in accordance with the rules of this code.” Therefore, for group 18, where *Nitratidesulfovibrio* and *Cupidesulfovibrio* collided, *Nitratidesulfovibrio* was established as the genus name of the group. In the same context, *Paradesulfovibrio bizertensis* of group 8 belongs to the same genus as *Desulfobaculum xiamenense* and is corrected as *Desulfobaculum bizertensis*. Despite the vacancy in the genus *Paradesulfovibrio*, group 1 deserves proposal as a new genus, and because they are of marine origin, we propose a new genus called *Salidesulfovibrio*.

From this study, objectivity and accuracy were obtained using indices, such as ANI, *d*DDH, and AAI, to compare genomic similarity and the classification was not based on phylogenetic analysis alone. As there are no clear standards for classifying the genus in the family Desulfovibrionaceae, an AAI cut-off value that did not incur a member included in multiple clusters for taxon delineation was defined to establish a clear standard. These accurately presented values will serve as a criterion to facilitate classification when new genera are added to this taxon.

Not all strains of the Desulfovibrionaceae family are extremophiles. However, strains belonging to this taxon have been reported to tolerate high heavy metal concentrations, and many are isolated from extreme marine environments such as hypersaline environments, deep sea sediment, and hydrothermal vent fields (with high metal concentration), and extreme terrestrial environments, such as regions contaminated with uranium, oil, and heavy metals. Because we noticed the frequent detection of this group in metal rich conditions, we explored the distribution of metal resistance-related genes through macroscopic *in silico* analysis. The analysis based on the genomes of Desulfovibrionaceae from the public database alone does not define characteristic of each group, yet it reveals a clear tendency within the given data. Based on taxa isolated from the biotic environment showed relatively low gene abundance and that evolutionarily close taxa were located closely on the PCA plot, it could be inferred that each genus showed a uniquely distinct gene abundance pattern under the influence of both environmental and evolutionary factors.

## Taxonomic Reclassifications

**Description of *Alkalidesulfovibrio* gen. nov.**

*Alkalidesulfovibrio* [Al.ka.li.de.sul.fo.vi’.bri.o. N.L. n. *alkali* (from Arabic article *al* the; Arabic n. *qaliy* ashes of saltwort) *alkali*; N.L. masc. n. *Desulfovibrio* a bacterial genus; N.L. masc. n. *Alkalidesulfovibrio* a *Desulfovibrio* living in alkaline environment].

Cells are vibrio-shaped, 0.5–0.8 × 1.4–1.9 μm. DNA G + C content is 64.5 mol%. Cells are motile by a single polar flagellum. The member of this genus shows anaerobic respiration, but tolerates short exposure to oxygen. Fermentative growth is observed. Sulfate, thiosulfate, and sulfite serve as electron acceptors and are reduced to sulfide. H_2_/CO_2_ and formate can serve as an electron donor in the presence of yeast extract or acetate. Thermotolerant, the optimum temperature for growth is 43°C and alkaliphilic, the optimum pH for growth is 9.0–9.4. The major menaquinone is MK-7. Desulfoviridin is present. The type species is *Alkalidesulfovibrio alkalitolerans.*

**Description of *Alkalidesulfovibrio alkalitolerans* comb. nov.**

Basonym: *Desulfovibr*io *alkalitolerans* (Abildgaard et al., 2006).

Other synonym: *Desulfohalovibrio alkalitolerans* (Spring et al., 2019).

The description is the same given by Abildgaard et al. (2006). The type strain is DSM 16529^T^ (= RT2^T^ = JCM 12612^T^). Genome and 16S rRNA sequence accession number: NZ_ATHI00000000 and AY649785.

**Description of *Salidesulfovibrio* gen. nov.**

*Salidesulfovibrio* [Sa.li.de.sul.fo.vi’.bri.o. L. masc. n. *sal* (*gen. salis*), salt; N.L. masc. n. *Desulfovibrio* a bacterial genus; N.L. masc. n. *Salidesulfovibrio* a *Desulfovibrio* living in saline environment].

Cells are rod or vibrio shaped, 0.3–0.5 × 1.0–4.0 μm with motility. Lactate and pyruvate are used as electron donors and some members show fermentative growth. When yeast extract and cysteine or acetate are present, H_2_/CO_2_ and formate can be used. Sulfate can be reduced to sulfide, and some members can use sulfur and Fe(III) as electron acceptors. Mesophilic, the optimum temperature for growth is 30–40°C and neutrophilic, the optimum pH for growth is 6.5-7.6. The DNA G + C content is 55.8–61.0 mol%. The type species is *Salidesulfovibrio onnuriiensis*.

**Description of *Salidesulfovibrio onnuriiensis* comb. nov. nom. rev.**

Basonym: *‘Paradesulfovibrio onnuriensis’* (Kim et al., 2020).

The description is the same given by Kim et al. (2020). The type strain is IOR2^T^ (= KCTC 15845^T^ = MCCC 1K04559^T^). Genome and 16S rRNA sequence accession number: CP040751.1 and MK968309.

**Description of *Salidesulfovibrio brasiliensis* comb. nov.**

Basonym: *‘Desulfovibrio brasiliensis’* (Warthmann et al., 2005).

The description is the same given by Warthmann et al. (2005). The type strain is JCM 12178^T^ (= DSM 15816^T^ = LVform1^T^). Genome and 16S rRNA sequence accession number: NZ_BBCB01000000 and AJ544687.

**Description of *Salidesulfovibrio halophilus* comb. nov.**

Basonym: *Desulfovibrio halophilus* (Caumette et al., 1991).

Other synonym: *Pseudodesulfovibrio halophilus* (Waite et al., 2020).

The description is the same given by Caumette et al. (1991). The type strain is DSM 5663^T^ (= ATCC 51179^T^ = SL8903^T^). Genome and 16S rRNA sequence accession number: SRX1760576 and X99237.

**Description of *Salidesulfovibrio senegalensis* comb. nov.**

Basonym: *Desulfovibrio senegalensis* (Thioye et al., 2017).

Other synonym: *Pseudodesulfovibrio senegalensis* (Galushko and Kuever, 2019), ‘*Paradesulfovibrio senegalensis*’ (Kim et al., 2020).

The description is the same given by Thioye et al. (2017), Galushko and Kuever (2019), and Kim et al. (2020). The type strain is DSM 101509^T^ (= BLaC1^T^ = JCM 31063^T^). Genome and 16S rRNA sequence accession number: NZ_WAIE00000000 and KT767981.

**Emended Description of the Genus *Psychrodesulfovibrio*** Galushko and Kuever, 2020d

*Psychrodesulfovibrio* [Psy.chro.de.sul.fo.vi’bri.o. Gr. masc. adj. *psychros*, cold; N.L. masc. n. *Desulfovibrio*, a bacterial genus; N.L. masc. n. *Psychrodesulfovibrio*, a *Desulfovibrio* living in the cold].

Cells are vibrio-shaped. DNA G + C content is 56.7-59.3 mol%. Cells are motile by a single polar flagellum. Strictly anaerobic with respiratory metabolism type. Sulfate serves as an electron acceptor. Succinate, malate, fumarate, pyruvate, lactate, and ethanol serve as electron donors with sulfate. Mesophilic, the optimum temperature for growth is 30-36^O^C and neutrophilic, the optimum pH for growth is 7.2-9.0. The type species is *Psychrodesulfovibrio psychrotolerans*.

**Description of *Psychrodesulfovibrio subterraneus* comb. nov.**

Basonym: *Desulfovibrio subterraneus* (Ueno et al., 2021).

The description is the same given by Ueno et al. (2021). The type strain is HN2^T^ (= DSM 101010^T^ = NBRC 112213^T^). Genome and 16S rRNA sequence accession number: NZ_BLVO00000000 and LC531930.

**Description of *Desulfobaculum bizertensis* comb. nov.**

Basonym: *Desulfovibr*io *bizertensis* (Haouari et al., 2006).

Other synonym: *Paradesulfovibrio bizentensis* (Waite et al., 2020).

The description is the same given by Haouari et al. (2006). The type strain is DSM 18034^T^ (= MB3^T^ = NCIMB 14199^T^). Genome and 16S rRNA sequence accession number: SRX1760595 and DQ422859.

**Description of *Nitratidesulfovibrio liaohensis* comb. nov.**

Basonym: *Cupidesulfovibrio liaohensis* (Wan et al., 2021).

The description is the same given by Wan et al. (2021). The type strain is XJ01^T^ (= DSM 107637^T^ = CGMCC 1.5227^T^). Genome and 16S rRNA sequence accession number: NZ_VSMK00000000.1 and MK260014.

## Data Availability Statement

The datasets presented in this study can be found in online repositories. The names of the repository/repositories and accession number(s) can be found in the article/Supplementary Material.

## Author Contributions

M-JP conducted every computational analysis and wrote manuscript. YK and KK designed experiment and instructed manuscript. MP helped programming for data treatment and repetitive calculation process. JY, TN, and Y-RR conducted literature study and organized data. All authors contributed to the article and approved the submitted version.

## Conflict of Interest

The authors declare that the research was conducted in the absence of any commercial or financial relationships that could be construed as a potential conflict of interest.

## Publisher’s Note

All claims expressed in this article are solely those of the authors and do not necessarily represent those of their affiliated organizations, or those of the publisher, the editors and the reviewers. Any product that may be evaluated in this article, or claim that may be made by its manufacturer, is not guaranteed or endorsed by the publisher.

## Footnotes

1
https://github.com/nanoporetech/medaka

## Supplementary Material

Supplementary Figure 1 Repetitive clustering and evaluation. Repetitive clustering and evaluation designed to find an appropriate AAI classification value without inclusion of a member in multiple cluster. Subjective intervention was avoided by repetitive clustering and evaluation with gradual increment of cut-off parameter to establish unbiased criteria, not setting strict borders after dividing the genus first.

Supplementary Figure 2 Correlation between AAI and POCP. Red dots represent values between inter-genus strains, and blue dots represent values between intra-genus strains. The color of regression equation in the upper left corresponds to the color of each dot.

Supplementary Figure 3 ANI and *d*DDH from pairwise whole-genome comparisons. The values of ANI and *d*DDH were expressed as heatmap. ANI is the lower left triangle indicated in violet color, and *d*DDH is the upper right triangle indicated in red color. Groups identified as the same species based on ANI and *d*DDH values are surrounded by black squares.

Supplementary Figure 4 Comparison of the RSCU data of the 27 groups. This figure verifies that there is a distinguishable difference in codon usage between each group. A line plot showing the average value of RSCU for each group.

Supplementary Figure 5 PCA results for metal resistance related gene abundance using z-score normalization. This figure verifies that there is a distinguishable difference in pattern of metal resistance related genes between each group.

Supplementary Table 1 Isolation information and genomic indices by bacterial strains.

Supplementary Table 2 The universal protein markers using for multi locus sequence alignments.

Supplementary Table 3 AAI values between genomes.

Supplementary Table 4 POCP values between genomes.

Supplementary Table 5 ANI values between genomes.

Supplementary Table 6 *d*DDH values between genomes.

Supplementary Table 7 RSCU values of each genome.

Supplementary Table 8 Relative abundance of metal related genes.
