# Supplementary material for: Establishment of Genome Based Criteria for Classification of the Family Desulfovibrionaceae and Proposal of Two Novel Genera, Alkalidesulfovibrio gen. nov. and Salidesulfovibrio gen. nov
Source: Front Microbiol. 2022 May 25;13:738205. doi: 10.3389/fmicb.2022.738205 (PMC9174804; doi:10.3389/fmicb.2022.738205)
Supplement: Supplementary Figure 1 — Repetitive clustering and evaluation. Repetitive clustering and evaluation designed to find an appropriate AAI classification value without inclusion of a member in multiple cluster. Subjective intervention was avoided by repetitive clustering and evaluation with gradual increment of cut-off parameter to establish unbiased criteria, not setting strict borders after dividing the genus first. [file Data_Sheet_2.PDF]

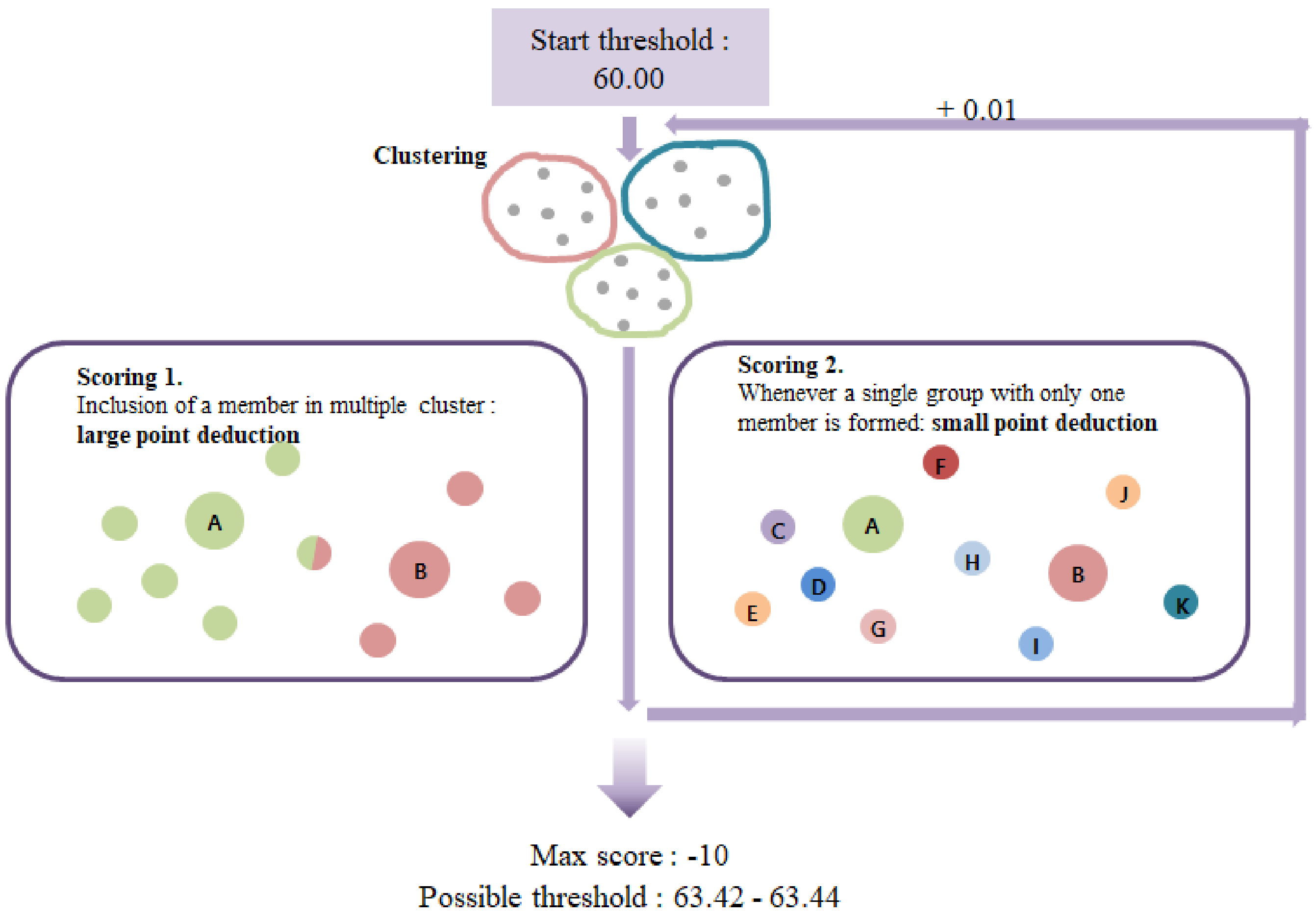

Supplementary Fig. 1. Repetitive clustering and evaluation
Repetitive clustering and evaluation designed to find an appropriate AAI classification value without inclusion of a member in multiple cluster. Subjective intervention was avoided by repetitive clustering and evaluation with gradual increment of cut-off parameter to establish unbiased criteria, not setting strict borders after dividing the genus first.

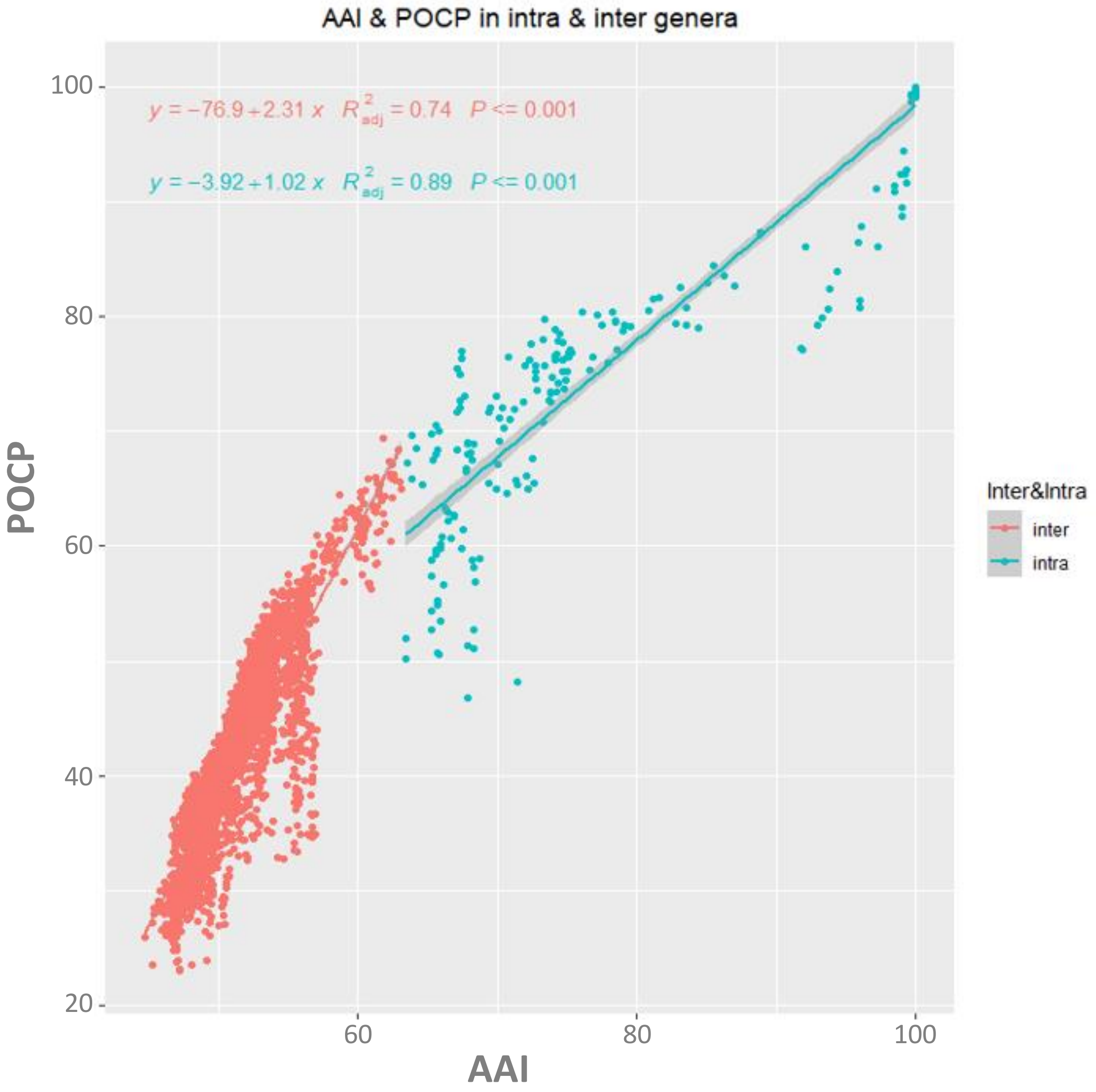

Supplementary Fig. 2. Correlation between AAI and POCP
Red dots represent values between inter-genus strains, and blue dots represent values between intra-genus strains. The color of regression equation in the upper left corresponds to the color of each dot.

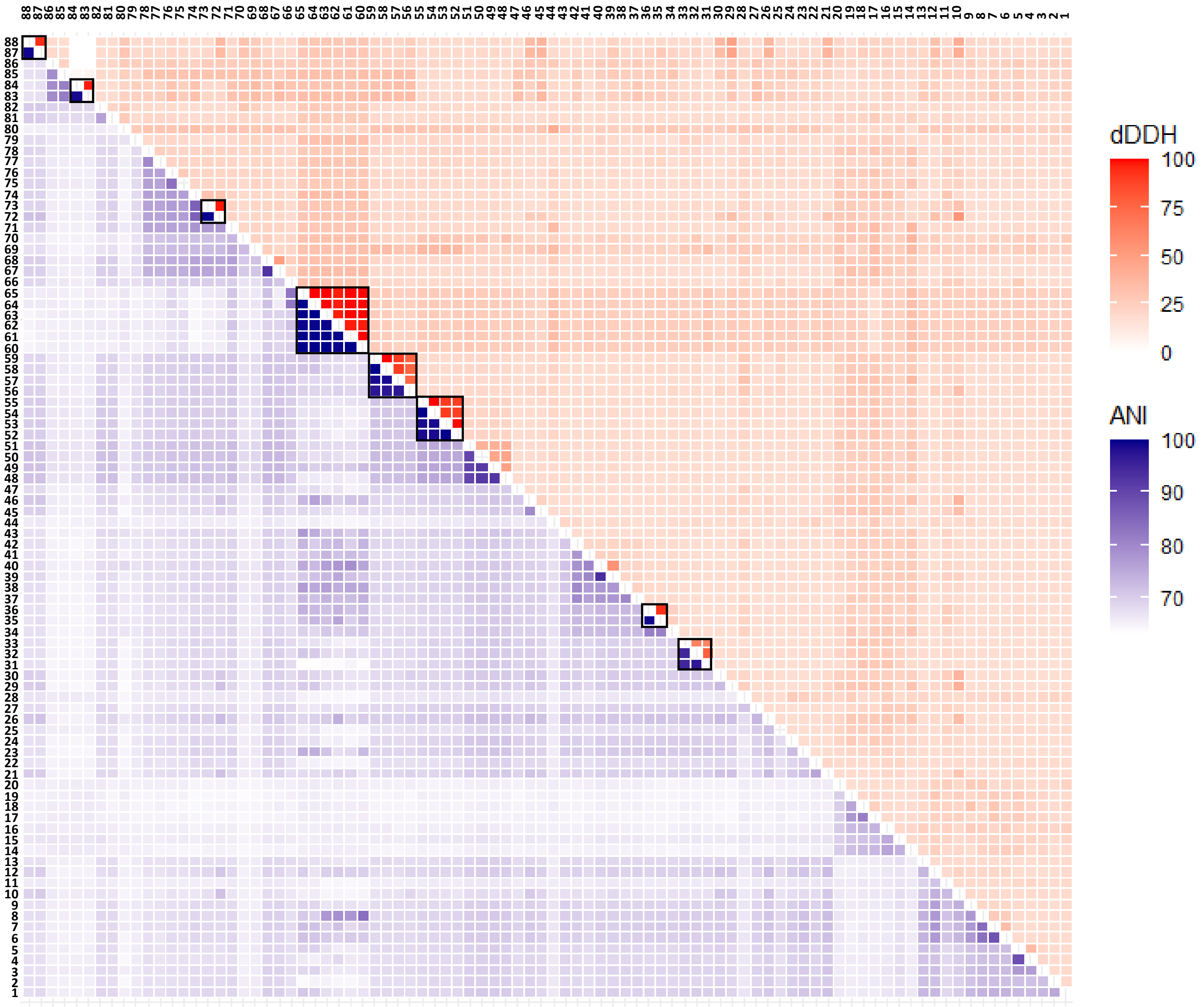

Supplementary Fig. 3. ANI and *d*DDH from pairwise whole-genome comparisons

The values of ANI and *d*DDH were expressed as heatmap. ANI is the lower left triangle indicated in violet color, and *d*DDH is the upper right triangle indicated in red color. Groups identified as the same species based on ANI and *d*DDH values are surrounded by black squares.

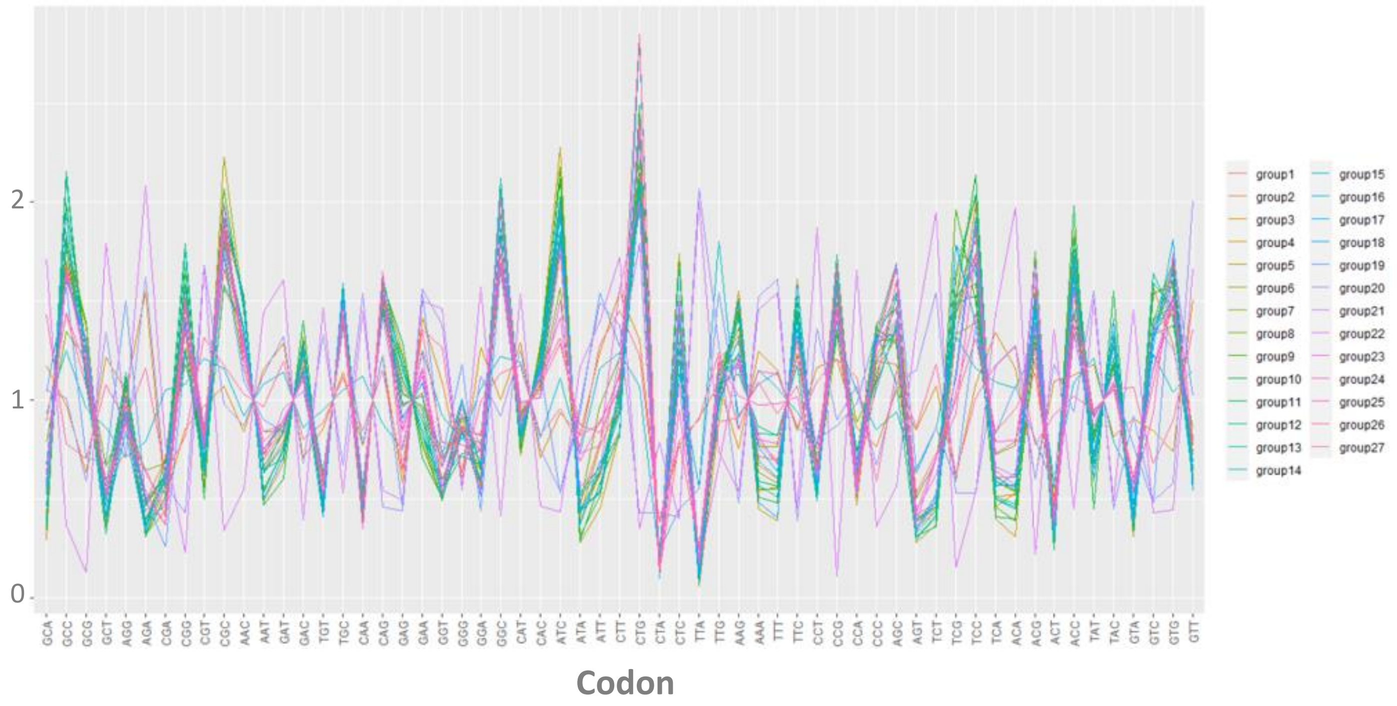

Supplementary Fig. 4. Comparison of the RSCU data of the 27 groups

This figure verifies that there is a distinguishable difference in codon usage between each group. A line plot showing the average value of RSCU for each group.

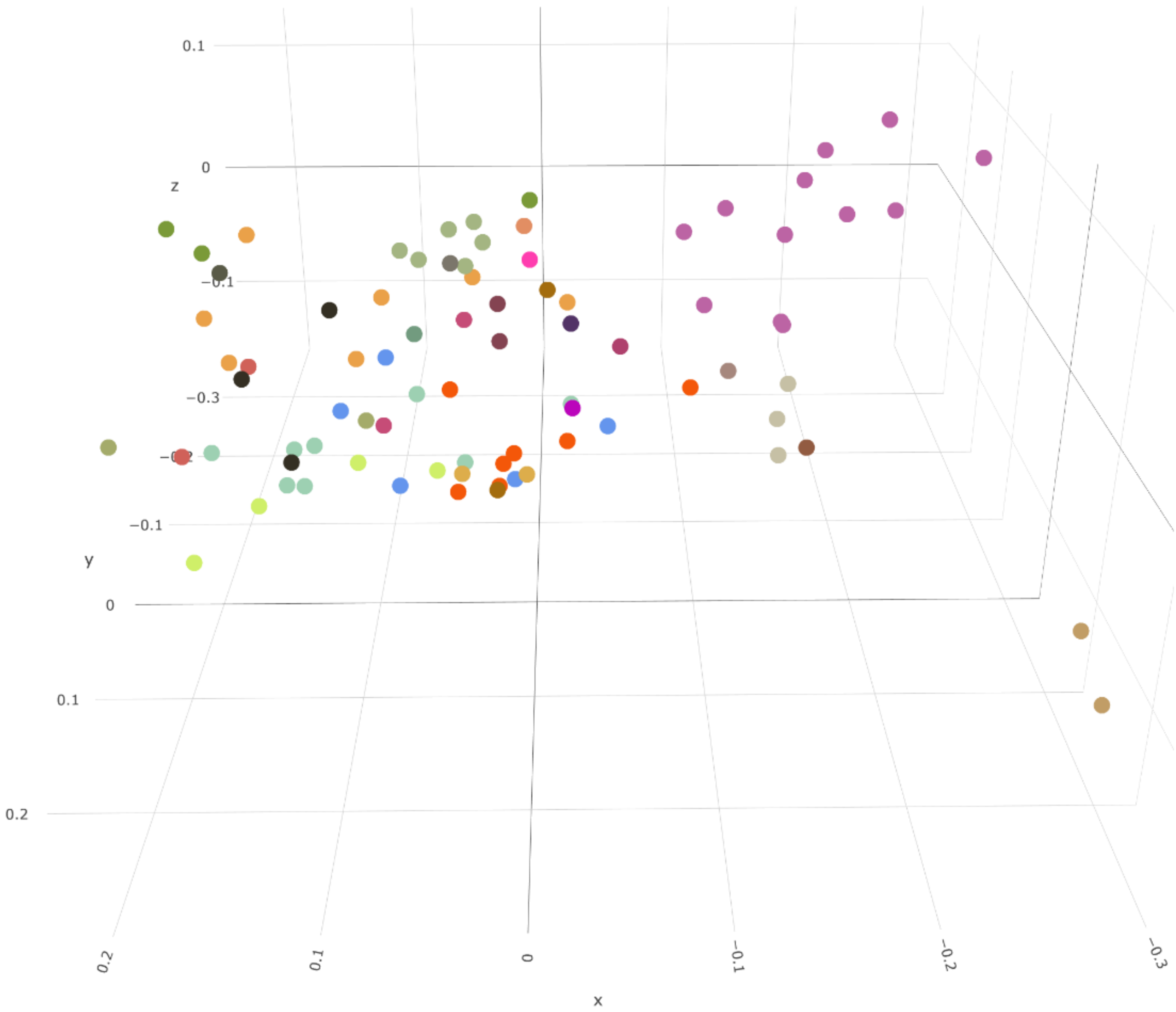

Supplementary Fig. 5. 3D plot of the three major axes generated by principal component analysis (PCA) using metal resistance related genes

This figure verifies that there is a distinguishable difference in pattern of metal resistance related genes between each group.
